# Supplementary material for: Atovaquone and Pibrentasvir Inhibit the SARS-CoV-2 Endoribonuclease and Restrict Infection In Vitro but Not In Vivo
Source: Viruses. 2023 Aug 30;15(9):1841. doi: 10.3390/v15091841 (PMC10534768; doi:10.3390/v15091841)
Supplement: Supplementary file 1 [file viruses-15-01841-s001.zip › viruses-2528125-supplementary.pdf]

Figure S1

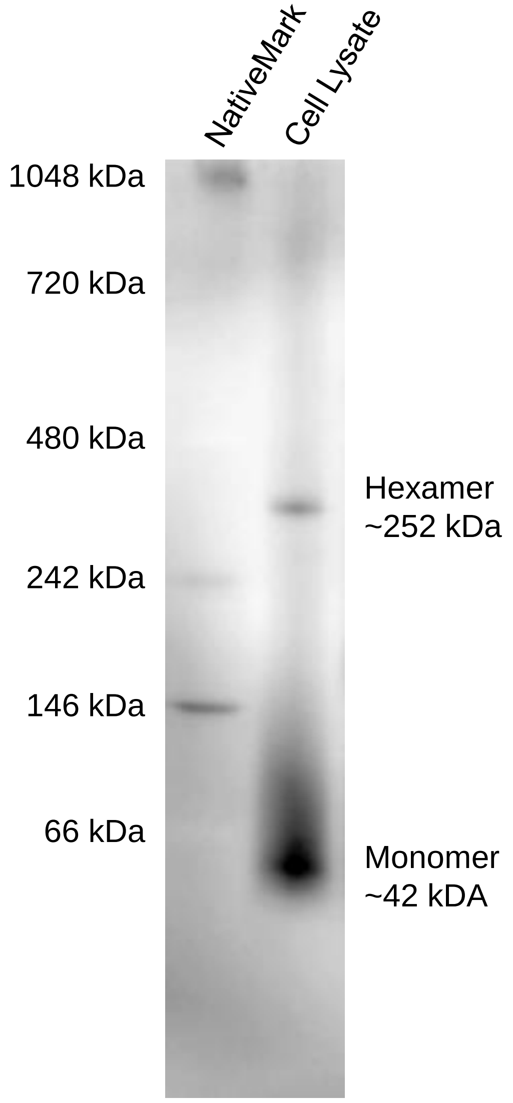

Supplemental Figure S1. Western blot analysis of HEK293T cells expressing strep-tagged nsp15 of SARS-CoV-2. HEK293T cells transfected with the pLVX-EF1alpha-SARS-CoV-2-nsp15-2xStrep-IRES-Puro were lysed in non-ionic detergent and separated under non-denaturing conditions. Protein bands specific to nsp15 and its hexameric oligomer were detected with polyclonal anti-Strep II tag antibody.

Figure S2

A

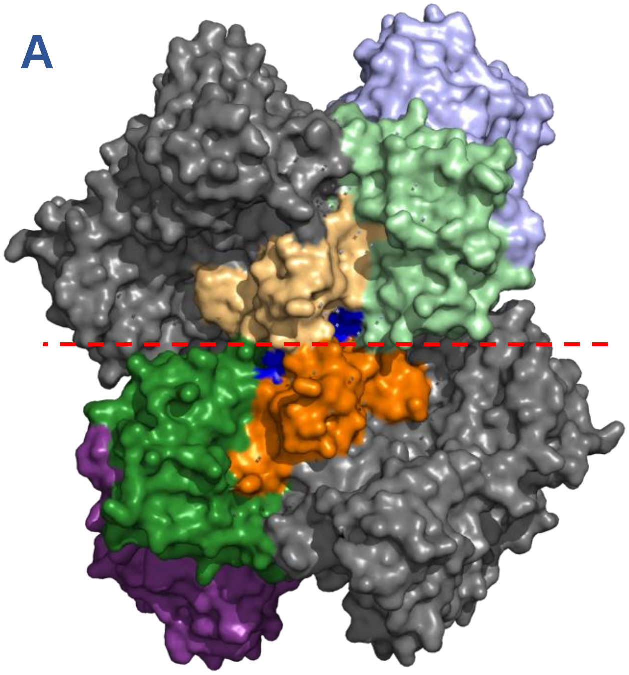

B

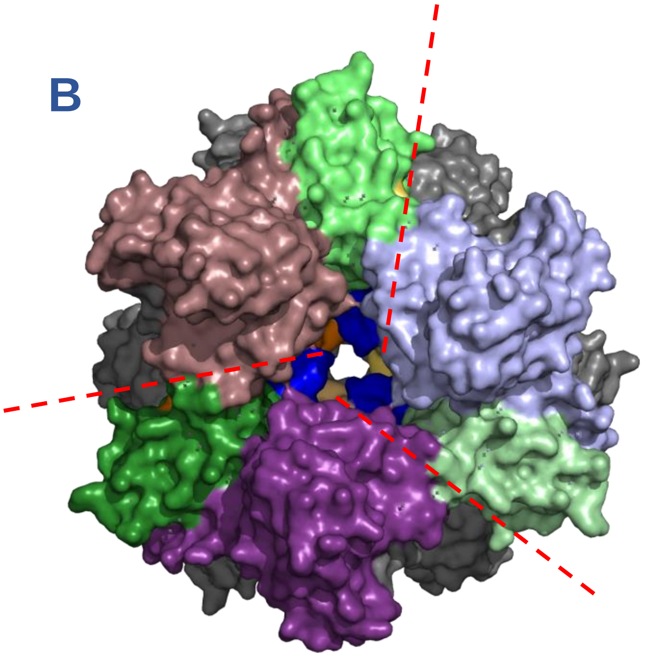

Supplemental Figure S2. Binding pockets of 11 drugs on hexameric nsp15 predicted by an in silico screening with FRAGSITE2. Binding pockets are highlighted in blue, while the oligomerization, middle, and catalytic domains are depicted in orange, green, and purple respectively. Domains from individual monomers forming a single interface appear in differing shades of the same base color, additional monomers appear in gray. (A) Binding pocket 1, predicted for oritavancin, ledipasvir, posaconazole, micafungin, linaclotide, pibrentasvir, desmopressin, and cyanocobalamin. Dashed line bisects opposing nsp15 trimers. The complete pocket is occluded by interactions between opposing trimers. (B) Binding pocket 2, predicted for rifamixin, rifapentine, and everolimus. Dashed lines intersect interfaces between individual monomers of one nsp15 trimer. Binding pockets remain exposed in the interior cavity of nsp15. Depictions generated based on PDB structure 7N06.

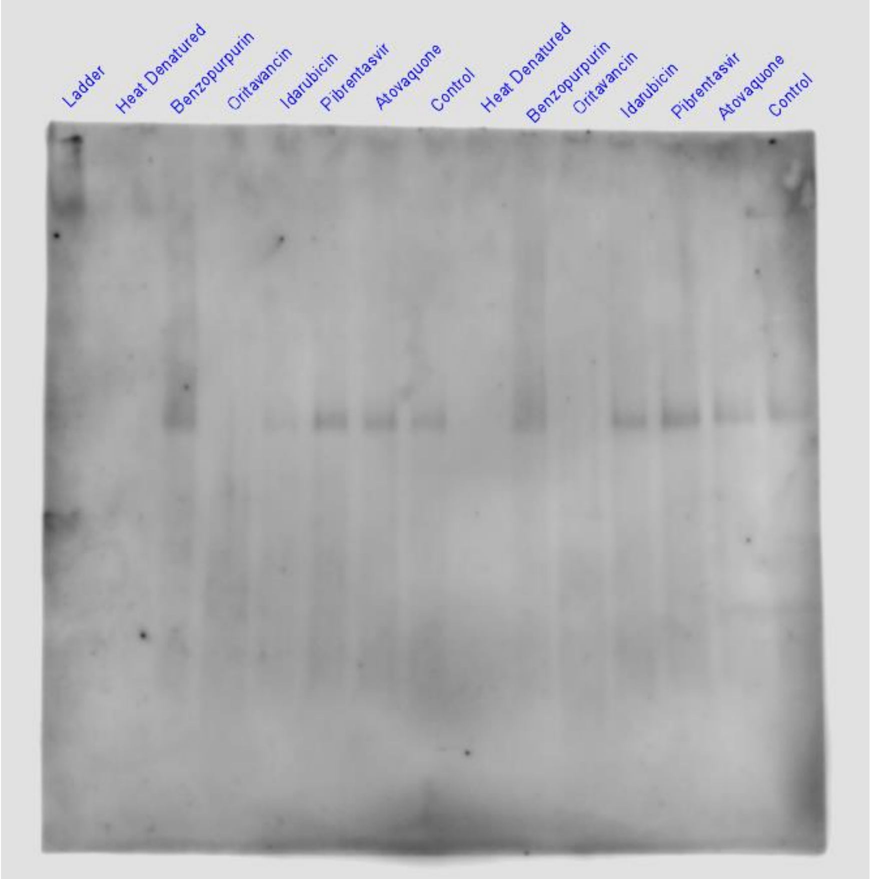

Supplemental Figure S3. Evaluation of drug-induced nsp15 aggregation. N-terminally his-tagged nsp15 was diluted to 700nM in NendoU Buffer containing 5mM $MnCl_2$ with or without 100μM of the indicated drug. Control and drug treated samples were then heated at 37°C for 10 minutes to simulate the nuclease assay reaction conditions. As a positive control, an additional preparation of nsp15 without drug was heated to 95°C for 10 minutes (Heat Denatured). Following incubation, the protein preparations were then separated on a non-denaturing polyacrylamide gel and evaluated by western blot. Control samples each produced a single band of the expected size corresponding to hexameric nsp15 (~240kDa), while the heat denatured samples produced no observable bands. Benzopurpurin, idarubicin, pibrentasvir, and atovaquone all produced a single band of similar size and staining intensity as control protein. However, oritavancin produced no detectable bands and appeared like the heat denatured aggregation positive control. Data presented is two of four repeats.

Lanes:

1. NativeMark Unstained Protein Standard
2. Heat denatured nsp15, replicate 1
3. Benzopurpurin treated nsp15, replicate 1
4. Oritavancin treated nsp15, replicate 1
5. Idarubicin treated nsp15, replicate 1
6. Pibrentasvir treated nsp15, replicate 1
7. Atovaquone treated nsp15, replicate 1
8. Control nsp15, replicate 1
9. Heat denatured nsp15, replicate 2
10. Benzopurpurin treated nsp15, replicate 2
11. Oritavancin treated nsp15, replicate 2
12. Idarubicin treated nsp15, replicate 2
13. Pibrentasvir treated nsp15, replicate 2
14. Atovaquone treated nsp15, replicate 2
15. Control nsp15, replicate 2

Figure S4

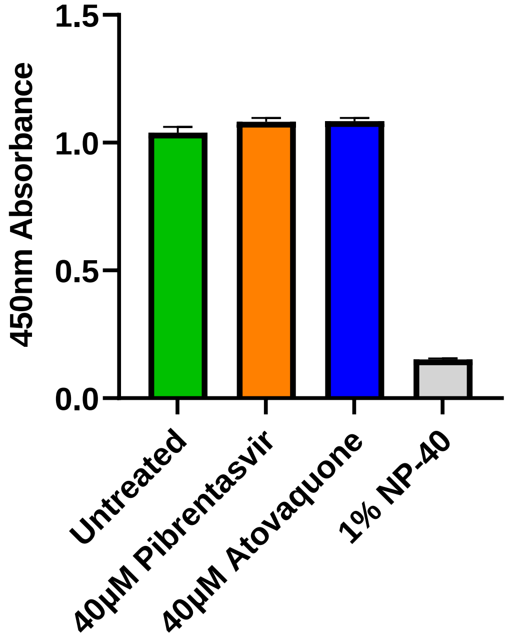

Supplemental Figure S4. MTS formazan formation viability assay. A549 wild-type cells were incubated for 24 hours with either control 1% FBS containing DMEM, or with the addition of 40μM atovaquone, 40μM pibrentasvir, or 1% NP-40. Metabolic activity was determined by addition of MTS reagent and production of the colored formazan product over a 1-hour period, measured by absorbance at 450nm. Results are representative of 1 experiment performed in triplicate.

Figure S5

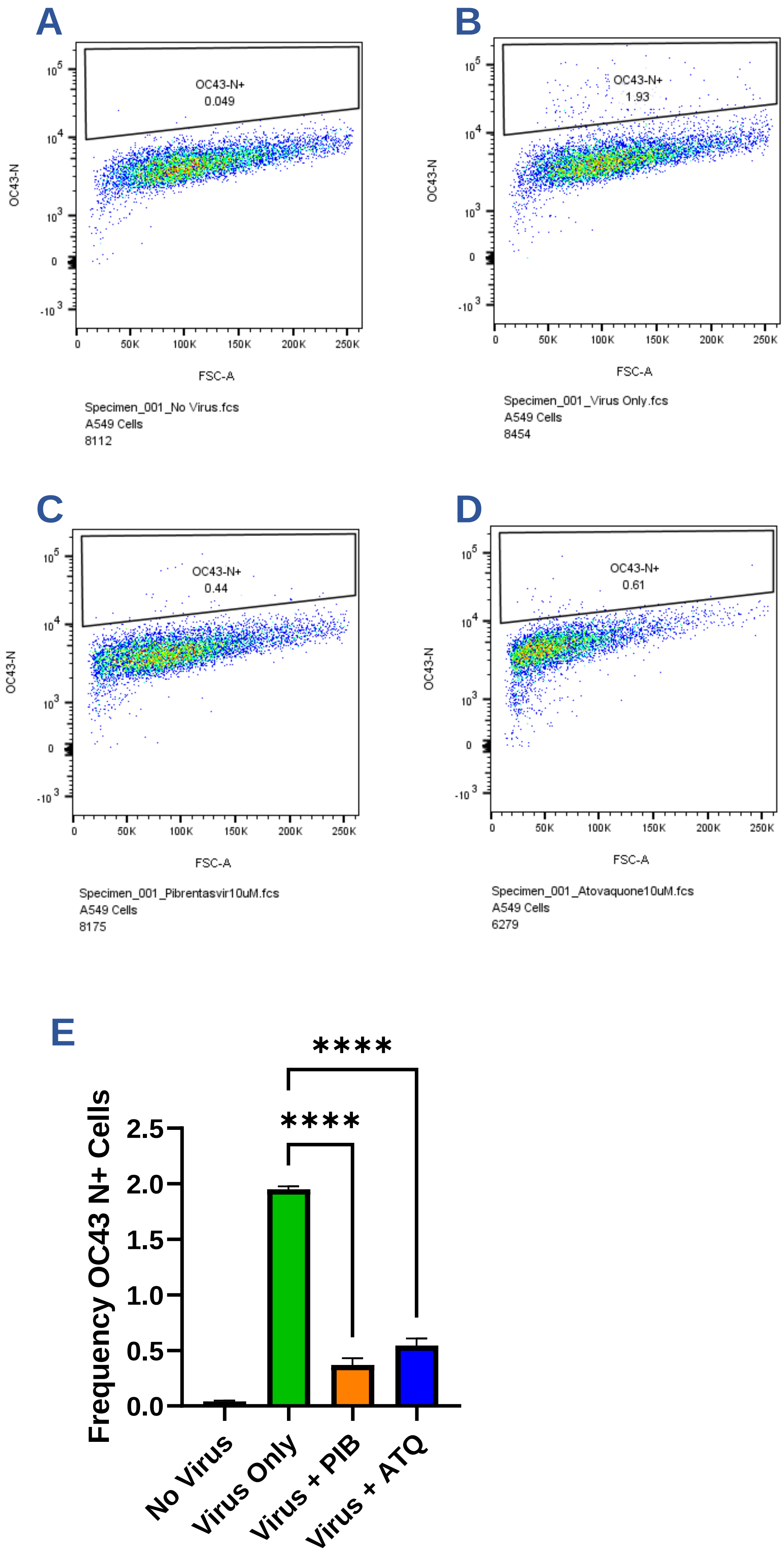

A
OC43-N+
0.049
OC43-N
FSC-A
Specimen_001_No Virus.fcs
A549 Cells
8112
B
OC43-N+
1.93
OC43-N
FSC-A
Specimen_001_Virus Only.fcs
A549 Cells
8454
C
OC43-N+
0.44
OC43-N
FSC-A
Specimen_001_Pibrentasvir10uM.fcs
A549 Cells
8175
D
OC43-N+
0.61
OC43-N
FSC-A
Specimen_001_Atovaquone10uM.fcs
A549 Cells
6279
E
Frequency OC43 N+ Cells
****
****
No Virus
Virus Only
Virus + PIB
Virus + ATQ

Supplemental Figure S5. Flow cytometric quantification of HCoV-OC43 infected cells with and without nsp15 inhibitor treatment. (A-D) Representative flow plots of A549 WT cells 12 hours post-infection with HCoV-OC43 at an MOI of 0.1. Inhibitors were added to a concentration of 10μM at 2 hours post-infection. At 12 hours post-infection, cells were trypsinized prior to fixation and permeabilization. HCoV-OC43 infected cells were detected by staining with primary mouse anti-HCoV-OC43 N protein and secondary Goat anti-mouse Ig conjugated to APC-Cy7. (A) Mock infected (B) HCoV-OC43 infected (C) HCoV-OC43 infected with 10μM pibrentasvir (D) HCoV-OC43 infected with 10μM atovaquone. (E) Graphical representation of flow plots presented in S2A. Columns and error bars are representative of 1 experiment performed in triplicate. Results analyzed by One-Way ANOVA with multiple comparisons. p-values represented as ****<.0001.
